# Integration Analysis of Pharmacokinetics and Metabolomics to Predict Metabolic Phenotype and Drug Exposure of Remdesivir

**DOI:** 10.3389/fphar.2021.779135

**Published:** 2022-01-05

**Authors:** Ping Du, Guoyong Wang, Ting Hu, Han Li, Zhuoling An

**Affiliations:** Department of Pharmacy/Phase I Clinical Trial and Research Unit, Beijing Chao-Yang Hospital, Capital Medical University, Beijing, China

**Keywords:** COVID-19, metabolomics, pharmacokinetics, remdesivir, drug exposure

## Abstract

Remdesivir has displayed pharmacological activity against SARS-CoV-2. However, no pharmacometabolomics (PM) or correlation analysis with pharmacokinetics (PK) was revealed. Rats were intravenously administered remdesivir, and a series of blood samples were collected before and after treatment. Comprehensive metabolomics profile and PK were investigated and quantitated simultaneously using our previous reliable HPLC-MS/MS method. Both longitudinal and transversal metabolic analyses were conducted, and the correlation between PM and PK parameters was evaluated using Pearson’s correlation analysis and the PLS model. Multivariate statistical analysis was employed for discovering candidate biomarkers which predicted drug exposure or toxicity of remdesivir. The prominent metabolic profile variation was observed between pre- and posttreatment, and significant changes were found in 65 metabolites. A total of 15 metabolites—12 carnitines, one N-acetyl-D-glucosamine, one allantoin, and one corticosterone—were significantly correlated with the concentration of Nuc (active metabolite of remdesivir). Adenosine, spermine, guanosine, sn-glycero-3-phosphocholine, and l-homoserine may be considered potential biomarkers for predicting drug exposure or toxicity. This study is the first attempt to apply PM and PK to study remdesivir response/toxicity, and the identified candidate biomarkers might be used to predict the AUC and C_max_, indicating capability of discriminating good or poor responders. Currently, this study originally offers considerable evidence to metabolite reprogramming of remdesivir and sheds light on precision therapy development in fighting COVID-19.

## Introduction

Coronavirus disease 2019 (COVID-19) caused by severe acute respiratory syndrome coronavirus 2 (SARS-CoV-2) is a serious threat for the global health environment (Zhu et al., 2020). As of 24 Nov 2021, a total of 258.16 million people had been infected with SARS-CoV-2 and 5,166,192 had died. In the face of the current global pandemic posed by SARS-CoV-2 infection, there is an urgent necessitation not only to prompt a fervent search for effective therapy but also to improve our knowledge of the metabolomic mechanism. Besides, even if this pandemic will be possibly controlled in the next few months, unexpected outbreaks and development of viral resistance to therapy due to virus mutations have exacerbated the already severe epidemic. As of now, several therapeutic strategies (e.g., small molecular chemical antiviral drug (Costanzo et al., 2020), traditional Chinese medicine (Ren et al., 2020), and vaccines (Lurie et al., 2020)) are being employed to improve the ratio of benefit/risk of patients with COVID-19.

Remdesivir, a nucleotide analog prodrug, which is metabolized into an analog of adenosine triphosphate (GS-441544, Nuc), has broad-spectrum activity against variety of viruses including Ebola, SARS-CoV-2, Middle East respiratory syndrome coronavirus (MERS-CoV), and COVID-19 (Lamb, 2020). Nowadays, remdesivir has been granted emergency use authorization by the U.S. Food and Drug Administration in November 2020 for hospitalized COVID-19 patients, and remdesivir may be considered a possible therapeutic option for COVID-19.

Metabolomics is one of the most powerful tools for studying the interaction between genetic background and exogenous and endogenous factors in human health. The concept of pharmacometabolomics (PM) was first illustrated in a study that showed metabolomics information in drug-free urine samples is predictive of both drug metabolism and toxicity of paracetamol (Clayton et al., 2006). Actually, PM can not only reveal the terminal metabolic profile by drug treatment but also reflect the metabolic status between tissues and fluids, which will be beneficial for understanding the biological mechanism of the disease (Kaddurah Daouk et al., 2015; Thomas et al., 2020). Unfortunately, the comprehensive metabolic mechanism of remdesivir was not fully figured out, especially with regard to metabolite reprogramming/perturbation.

Limited studies have been revealed for the metabolite changes among COVID-19 patient cohorts, such as cytosine and tryptophan–nicotinamide pathways (Blasco et al., 2020), lipids (Archambault et al., 2021), amino acids and fatty acids (Shen et al., 2020), and eicosanoids (Du et al., 2021a). However, to the best of our knowledge, no comprehensive metabolic profiling literature studies were reported pertaining to remdesivir treatment both *in vitro* and *in vivo*. It is therefore reasonable and feasible to study the association between metabolic profiles and remdesivir treatment. In view of the shortcomings described before, the purpose of this study was originally proposed to longitudinally and transversally investigate the metabolic fingerprint induced by remdesivir in rats. Furthermore, by means of several multivariate statistical analyses, an integration analysis of metabolomics and pharmacokinetics (PK) was employed in order to predict the metabolic phenotype and drug exposure. Overall, we reveal the first in-depth interrogation of trajectory changes that benefit propitious understanding of how remdesivir interacted with small molecular metabolites, and several candidate predictive biomarkers were investigated and validated for drug response or toxicity. The results of this study will shed light on how remdesivir disturbed the metabolic profiles and offered meaningful references for precision therapy in patients of COVID-19.

## Materials and Methods

### Chemicals

Both standards of metabolites and stable isotope-labeled internal standards (IS) were obtained from Sigma-Aldrich (St. Louis, MO, United States), Cayman Chemical (Ann Arbor, MI, United States), Bidepharm (Shanghai, China), Steraloids (Newport, RI, United States), Cambridge Isotope Laboratories (Cambridge, MA, United States), and Cayman Chemical or Steroids (Supplementary Table S1). Detailed information was given in our previous study (Hu et al., 2020a). Organic solutions (e.g., acetonitrile, isopropyl alcohol, and methanol) of HPLC grade were purchased from Fisher Scientific (Pittsburgh, PA, United States). The modifier of the mobile phase—formic acid—was obtained from Co., Inc. (Fairfield, OH, United States). Ultrapure Millipore water was prepared by a purification system.

### Experimental Animals and Metabolomics Profiling After Remdesivir Treatment

Animal experiments were carried out according to the Guidelines for the Care and Use of Laboratory Animals. Rats (*n* = 6, 6–8 weeks old, 180–220 g) were purchased from Beijing Vital River Laboratory Animal Technology Co., Ltd. (Beijing, China) and raised in controlled environment (25 ± 2°C, 40–70% humidity, and 12-h light on/off cycle). Rats were fed with free drinking water and standard feed. All rats were placed in a single rat IVIVC cage. Animals were accommodated for 1 week prior to the experiment.

For the longitudinal PM of remdesivir, the rats were intravenously administered remdesivir (5 mg/kg) dissolved with 12% sulfobutylether-β-cyclodextrin in water. Blood samples were collected from ophthalmic veins by sterile capillary into (NaF/K-Ox) tubes at before (0 h) and after administration (5, 15, and 30 min and 1, 2, 4, 8, 12, 24, and 48 h) and then directly centrifuged to obtain plasma (3,500 rpm, 10 min, 4°C). All plasma samples were retained at -80°C for further analysis.

For the transversal PM, raw metabolomic data were separated into two parts: before (pre-dose) and after (post-dose) administration. The metabolomic profiling and trajectory effect of remdesivir were investigated and analyzed using multivariate statistical analysis methods.

### HPLC-MS/MS System

High-performance liquid chromatography-tandem mass spectrometry system (HPLC-MS/MS, Spark Holland; API 5500, SCIEX, Canada) was adopted for targeted metabolomic analysis. The chromatography columns (Waters BEH, HSS T3) and elution solvent (gradient elution) were all evaluated and used according to our previous study (Hu et al., 2020a). The column temperature was set at 20°C with injection volume of 5 μL. Water phase and organic phase (acetonitrile:isopropyl alcohol = 7:2, *v/v*) contained 0.1% formic acid, and gradient elution was achieved within 27 min.

All analytes were detected *via* both negative and positive modes with the help of rapid polarity switching and the advanced MRM algorithm. The MS electrospray voltage was 4500 and 5500 V for negative or positive modes, respectively. The optimized MRM parameters are shown in Supplementary Table S1. Detailed parameters of HPLC-MS/MS are shown in our previous study (Hu et al., 2020a).

### Sample Preparation

The one-step protein precipitation method was adopted for this metabolomic analysis. Briefly, an aliquot of 50 μL plasma was spiked with 10 μL IS mixture (eight ISs, 400 ng/ml) and 140 μL precipitation solution (-20°C methanol). Afterward, the mixture was vortexed for 2 min and centrifuged at 13,500 rpm for 10 min at 4°C. The solutions mentioned earlier were injected into the HPLC-MS/MS system for analysis.

For the purpose of ensuring reliable quantitation of all analytes and better comparability in routine analysis, quality control (QC) samples were prepared by pooling equal volumes of unknown plasma. Briefly, six aliquots of pooled QC samples were constructed as real samples and the analytical sequence was interpolated to check the status of sample injection and the HPLC-MS/MS system.

### Pharmacokinetic Analysis

Pharmacokinetic analysis was performed as reported in our previous study (Du et al., 2021b). Briefly, chromatography separation (LC-20ADXR, Shimadzu, Japan) was accomplished on a Waters XBrige C_18_ column (50 × 2.1 mm, 3.5 μm) using gradient elution. The temperatures of the autosampler and column were set at room temperature and 40°C, respectively. The flow rate was kept at 0.4 ml/min under the gradient elution mode (Du et al., 2021b).

The mass spectrometry parameters (QTRAP 5500, SCIEX, Canada) were used, and the protonated molecule [M + H]^+^ ion was used for all analytes. The quantitative MRM was set at *m/z* 292.2→163.2 for Nuc and 237.1→194.1 for the IS (carbamazepine). The calibration curve was linear in the range of 2–1,000 ng/ml (Nuc, the active metabolite of remdesivir). A simple and high-throughput protein precipitation method was used for preparing plasma samples (Du et al., 2021b). Method validation of selectivity, sensitivity, accuracy, precision, recovery, matrix effect, stability, and incurred sample reanalysis met the criteria of method validation guidelines. Detailed results were illustrated in our previous study (Du et al., 2021b).

### Multivariate Statistical Analysis and Data Processing

Raw data files were processed and checked by MultiQuant 3.0.1 (SCIEX). The concentrations of analytes were calculated according to the calibration curve. Pharmacokinetic parameters, such as C_max_ (maximum concentration) and AUC (area under the curve) were calculated using Phoenix (Pharsight 8.3, Mountain View, CA) software. Pearson’s correlation was used to investigate the relation between metabolomics data and PK parameters using IBM SPSS 26.0 (Armonk, New York, United States). SIMCA-P software (v14.1, Umetric, Umeå, Sweden) was used to build mathematic models including unsupervised principal component analysis (PCA), supervised orthogonal projection to latent structures-discriminant analysis (OPLS-DA), and partial least squares (PLS). The compounds with values of variable importance in the projections (VIPs) > 1 and statistical significance of *p* < 0.05 were picked out for further identification and metabolic pathway analysis. Two hundred random permutation tests were used to check overfitting and random effects, which can assess the predictive ability of the model. Pathway analysis was achieved using online MetaboAnalyst 5.0 (http://www.metaboanalyst.ca), while the Kyoto Encyclopedia of Genes and Genomes (KEGG) database was also used for hierarchical cluster analysis (HCA) and the *t*-test and mechanism analysis. A *p* value less than 0.05 was considered statistically significant.

## Results

### Overview of Targeted Metabolomics for Remdesivir

**FIGURE 1:**
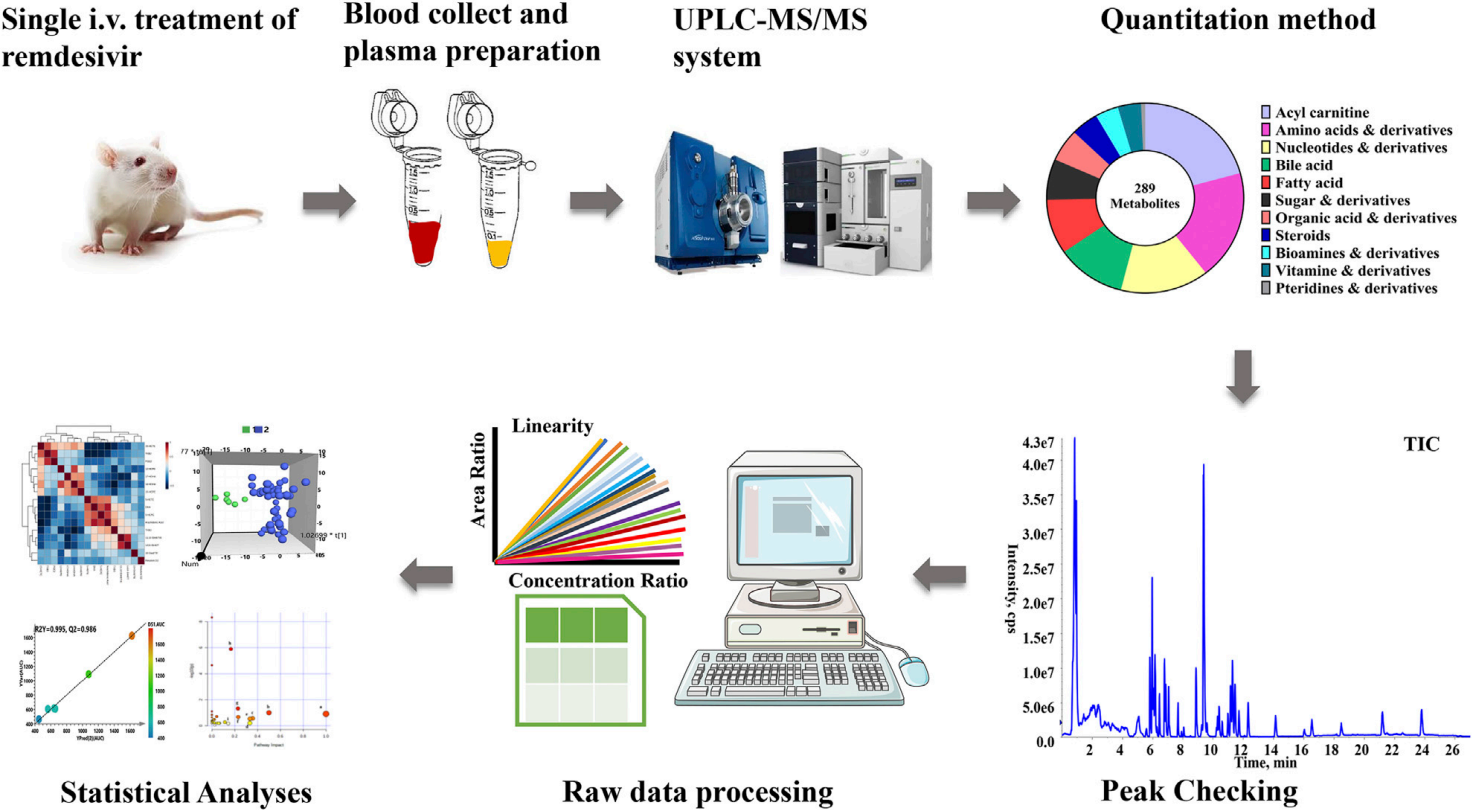
Workflow of targeted metabolomics applied in this study.

The comprehensive metabolomics method developed in our laboratory was utilized for present quantitation in plasma samples (Hu et al., 2020a). As described in Figure 1, a total of 289 metabolites, which contained amino acids, bile acids, and vitamins, were covered in the present metabolomics method. All biologically active metabolites can be quantitated during the 27-min analysis period. The calculation linearity ranged from 0.2 to 5,000 ng/ml, which provides powerful capability for successful quantitation of low-abundance compounds. Furthermore, other parameters were all carefully investigated (Supplementary Table S1).

### Longitudinal Metabolic Profiling Analysis

**FIGURE 2:**
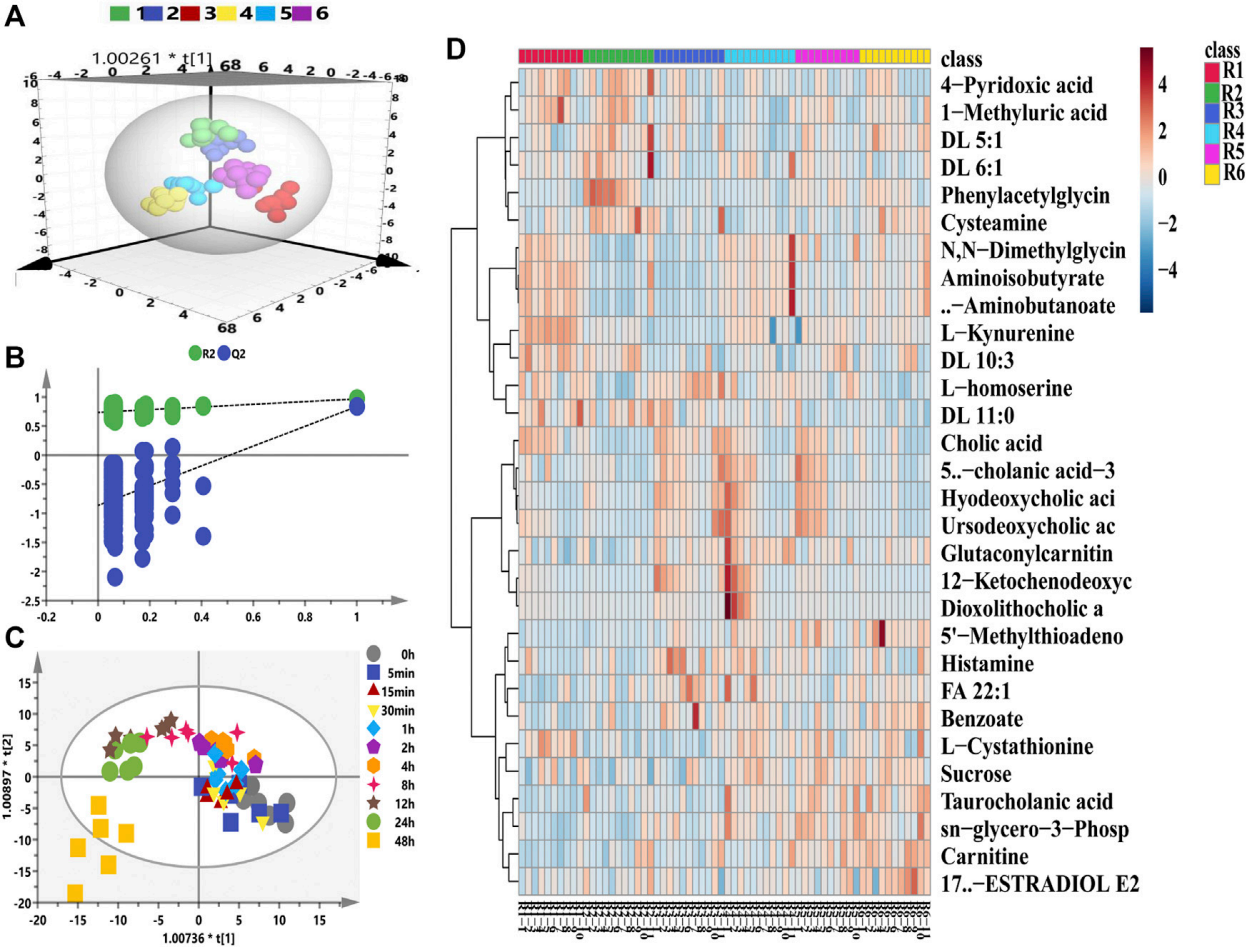
Longitudinal metabolomic fingerprints of remdesivir. **(A)** OPLS-DA score plots from six rats. **(B)** Random permutation test with 200 iterations. **(C)** Time-dependent trajectory of remdesivir-treated metabolites by OPLS-DA score plots. **(D)** Heatmap of differential metabolites ranked in the top 30.

With respect to the longitudinal metabolic fingerprints after remdesivir treatment, metabolite peak area ratios of all plasma samples were pooled into the statistical data set. For metabolic data quality analysis, a distance-to-model (DModX) plot was used to check the outliers, and all samples were in the limit of 2 (data not shown). Both PCA and OPLS-DA were used to integrate and co-analyze all data to explore the longitudinal metabolic trajectory in all rats. Individuals were utilized as the grouping basis, and dots of the same color represented samples of one rat at different time points (Du et al., 2021a). From the results of Figure 2A, plasma samples of one rat were divided into tight clusters, which indicated that the longitudinal metabolic fingerprint of the same rat was relatively stable after remdesivir treatment. The metabolic fingerprint changes generated by individuals were greater than the metabolic disturbance induced by remdesivir treatment. This model was validated by 200-time permutation, and no overfitting was observed (Figure 2B, *R* ^2^= (0.0, 0.743), Q^2^= (0.0, −0.835)). HCA was calculated based on the Euclidean correlation with the Ward clustering algorithm (Figure 2D). In order to analyze the metabolic trajectory at different treated time points, the metabolic changes between pre-dose and post-dose samples (5, 15, 30 min, 1, 2, 4, 8, 12, 24, and 48 h) were analyzed and are shown in Figure 2C. The metabolic trajectory was almost steady from 5 min to 24 h; however, distinguished metabolic profiles were shown for that at 48 h. Besides, the number of VIP >1.0 was 80, and the highest VIP was phenylacetylglycine (1.97), l-kynurenine (1.87), and cholic acid (1.83), respectively (Supplementary Figure S1).

### Transversal Metabolomics of Remdesivir

**FIGURE 3:**
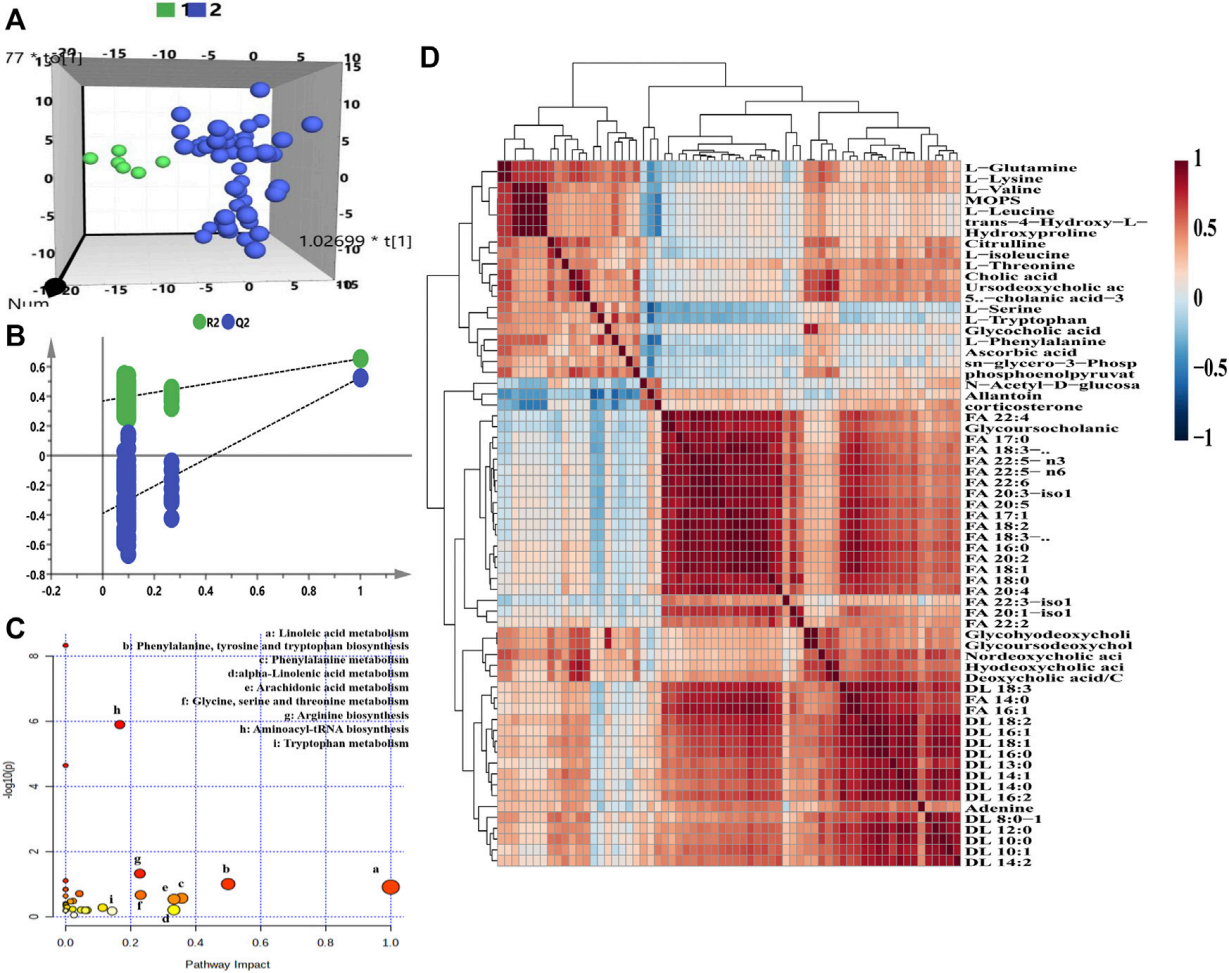
Transversal metabolomics fingerprint of remdesivir before and after treatment. **(A)** OPLS-DA score plot. **(B)** Random permutation test with 200 iterations. **(C)** Overview of pathway enrichment analysis of the altered metabolites between pre- and post-dose. **(D)** HCA of metabolite–metabolite correlation in response to remdesivir treatment.

For the purpose of exploring the metabolic phenotype variation caused by remdesivir treatment, the metabolomics features at baseline (pre-dose) were compared with those at the treated period (post-dose). For the transversal PM of remdesivir, Figure 3A illustrated that all metabolic data were introduced for OPLS-DA. Although limited metabolic samples were used in the present study at baseline time points, the individuals of both groups were discriminated well in the OPLS-DA model. Moreover, the random permutation test with 200 iterations was performed to investigate the validity and predictability of the OPLS-DA model. Figure 3B shows that no overfitting was observed for all introduced data (*R* ^2^= (0.0, 0.354), Q^2^= (0.0, -0.424)). The most significantly changed metabolites between pre- and post-dose were picked up using the independent samples *t*-test. A total of 65 metabolites were observed to be significantly changed with the cutoff of VIP >1, *p* < 0.05 (Supplementary Table S2). The results of pathway analysis indicated that the high-impact pathways were linoleic acid metabolism; phenylalanine, tyrosine, and tryptophan biosynthesis; phenylalanine metabolism; alpha-linolenic acid metabolism; arachidonic acid metabolism; glycine, serine, and threonine metabolism; and arginine biosynthesis. As shown in Figure 3C, although the impact of linoleic acid metabolism was the highest, neither the hits nor the *p* value met the acceptable criteria. After pathway analysis, two pathways, arginine biosynthesis (two hits) and aminoacyl-tRNA biosynthesis (nine hits), were considered the disturbance metabolism pathway, with impact of 0.228 and 0.167 and *p* value of 0.046 and 1.26 E-06. The arginine biosynthesis pathway consisted of l-citrulline and l-glutamine; the aminoacyl-tRNA biosynthesis pathway comprised nine amino acids (l-phenylalanine, l-glutamine, l-serine, l-valine, l-lysine, l-isoleucine, l-leucine, l-threonine, and L-tryptophan) (Supplementary Table S3).

As shown in Figure 3D, the significantly changed metabolites correlated with each other positively or negatively. Taken together, inherent metabolic phenotype variations had taken place as a result of the treatment of remdesivir.

**FIGURE 4:**
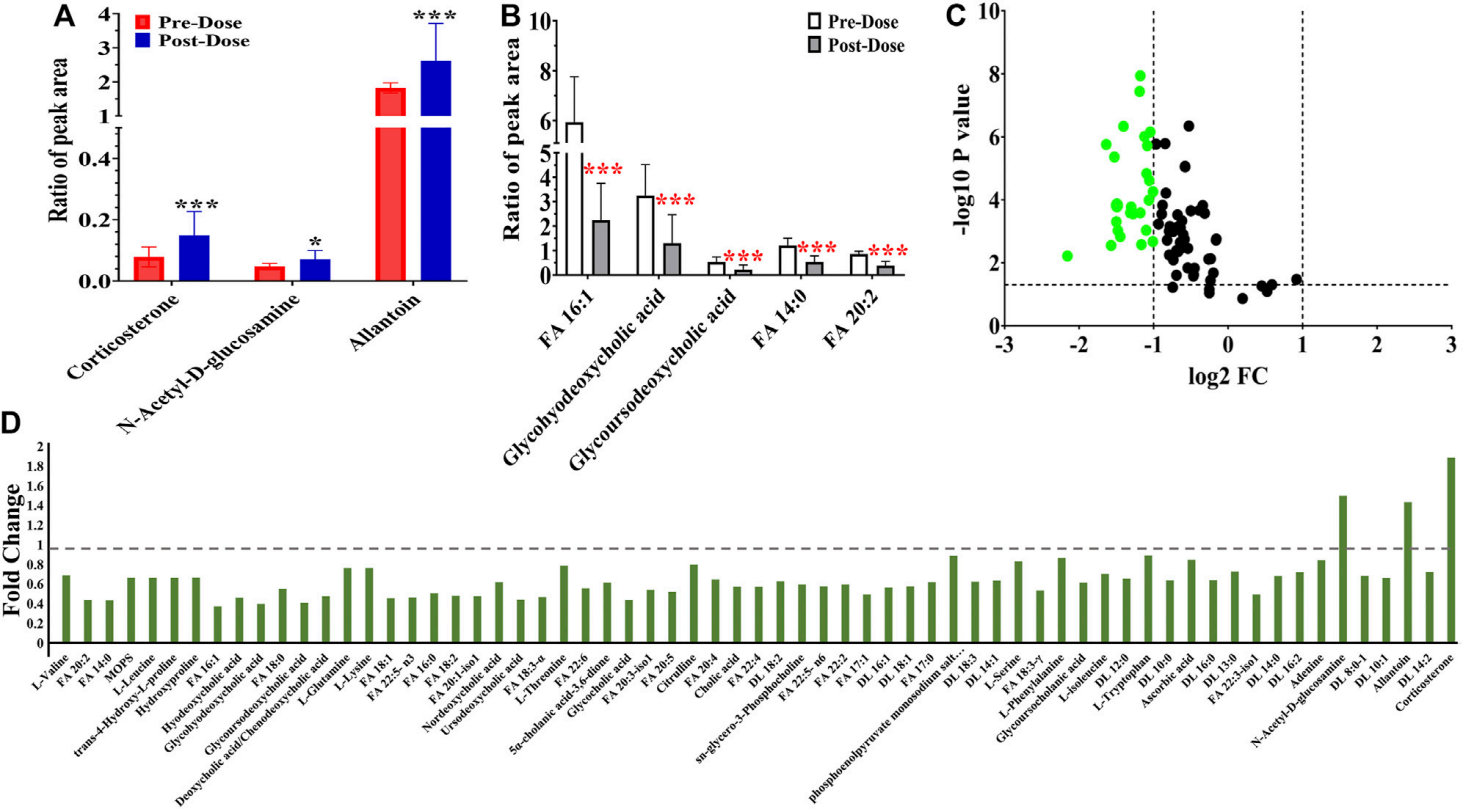
Metabolic peak area ratio comparison between the pre- and post-dose. **(A)** Upregulated metabolites compared with the pre-dose group. **(B)** Most significant downregulated metabolites compared with the pre-dose group. **(C)** Volcano map of metabolites with VIP >1, *p* < 0.05, green dots represent metabolites of FC < 0.5. **(D)** Fold change landscape of all significant disturbance metabolites. *p < 0.05 and ***p < 0.001, two-tailed unpaired *t*-test.

Additionally, the metabolic intensity of 65 significantly changed metabolites was compared transversally. The differentially regulated metabolites are presented by fold change (FC > 1: upregulated metabolites; FC < 0.5: downregulated metabolites). The upregulated results (three metabolites) are shown in Figure 4A. Besides, the intensity of the remaining 62 metabolites was downregulated, and the five most significantly downregulated metabolites included two fatty acids, glycohyodeoxycholic acid and glycoursodeoxycholic acid compared between pre- and post-dose treatment (Figure 4B). Figure 4C of the volcano map indicates that 41.54% (27/65) metabolites whose FC < 0.5 were thought to be significantly disturbed after remdesivir treatment.

### Correlation Analysis Between Metabolomics and Pharmacokinetics

**FIGURE 5:**
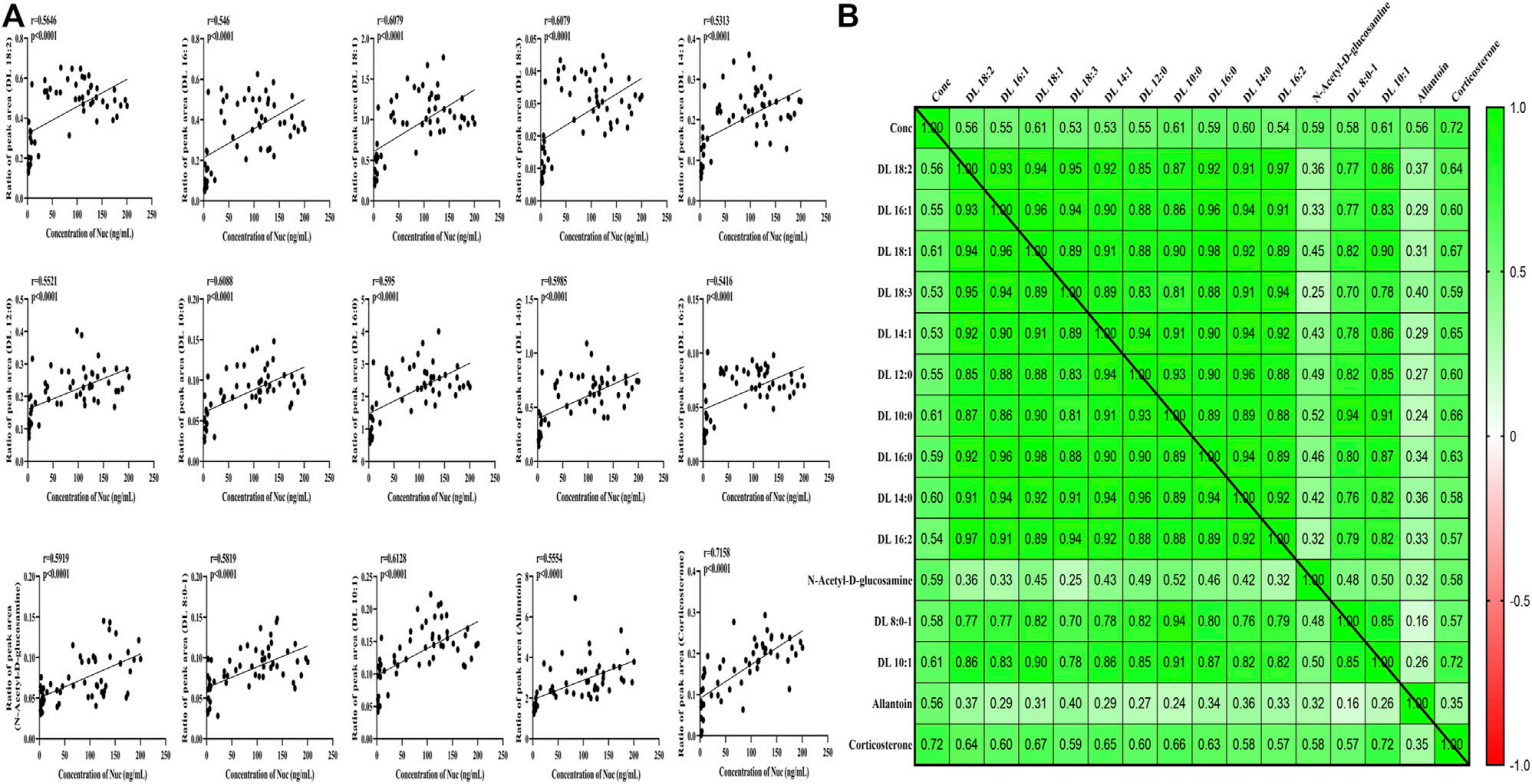
Correlation analysis between significantly changed metabolites and concentration of Nuc. **(A)** Correlation values. **(B)** Heat map of disturbed metabolites.

To further explore whether metabolic fingerprint disturbance accompanies the plasma drug exposure, Pearson’s correlation analysis was conducted to determine what metabolites are highly interplayed with this tendency. As shown in Figure 5, a total of 15 metabolites—12 carnitines, one N-acetyl-D-glucosamine, one allantoin, and one corticosterone—were significantly correlated with the concentration of Nuc (r > 0.5, *p* < 0.05).

The mean concentration-time curve and pharmacokinetic (PK) parameters of Nuc were elucidated in rats after intravenous administration of remdesivir (5 mg/kg) according to our previous study (Du et al., 2021b). The individual blood concentration-time curves of Nuc measured in six rats presented a high degree of interindividualized variation of PK behavior (Supplementary Figure S1). During the four parameters summarized in table, t_1/2_ variation (4.22-fold difference) was the most significant due to individual treatment among all rats. The variation fold change of C_max_, T_max_, and AUC_0-t_ was 1.64-, 2.00-, and 3.50-fold difference between the maximum and minimum values. It is known that AUC and C_max_ can be considered as indicators of drug efficacy or toxicity to some extent (Xing et al., 2019). Therefore, AUC and C_max_ were chosen for further PLS model analysis.

A supervised PLS model was constructed for capably predicting PK parameters and identifying relationship between two groups of variables (Xing et al., 2019). Thus, the 207 endogenous metabolites were described as one group of variables (*X*, the predictive variables), while AUC or C_max_ were represented as a group of variables (*Y*, the response variables), respectively. First, the PCA model was constructed to find out outliers to avoid deviation of prediction. All rats are distributed according to pre-dose metabolic profiles (Supplementary Figure S2).

**FIGURE 6:**
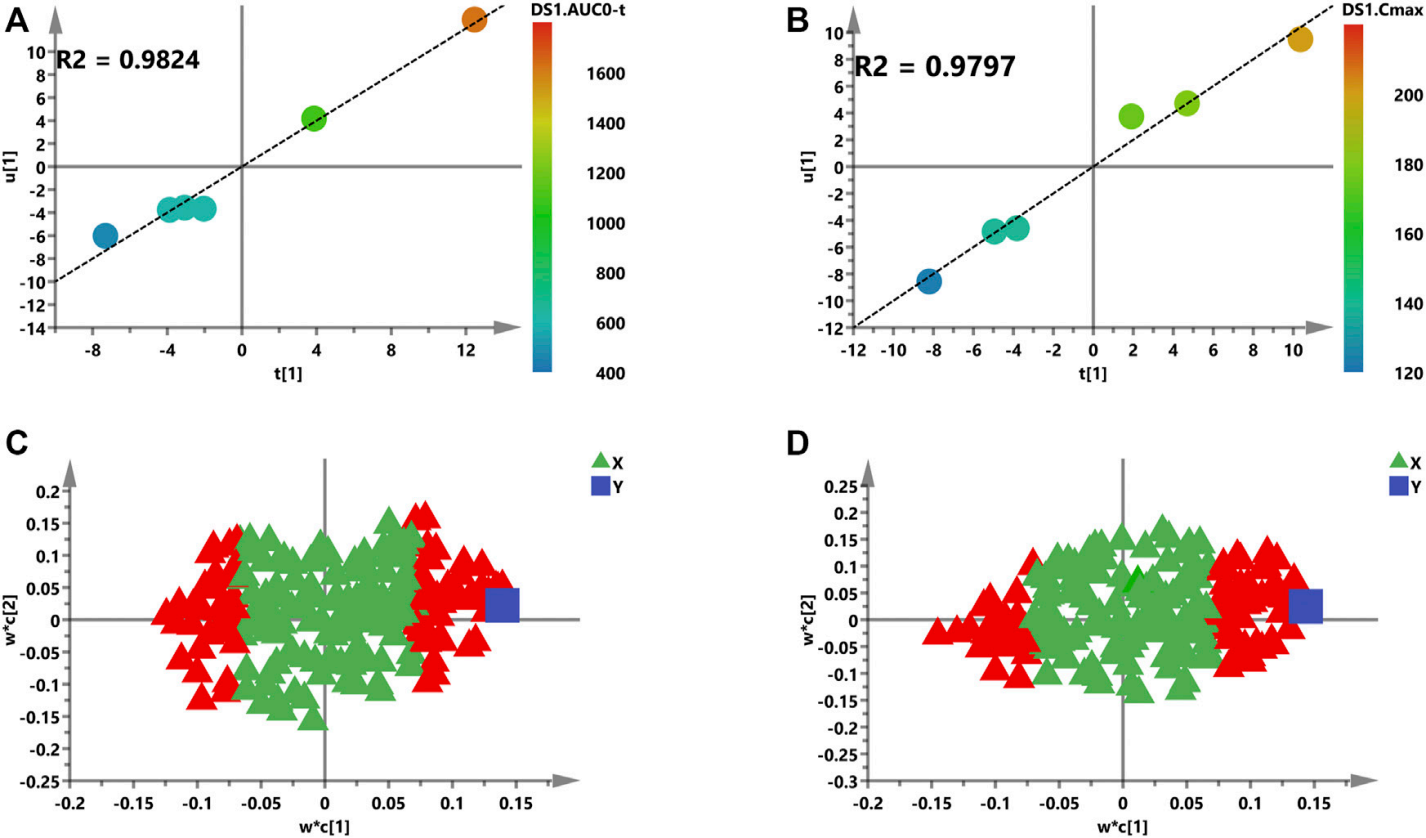
Initial PLS models of the pre-dose metabolic profile for predicting PK parameters of Nuc. **(A)** and **(B)** are score plots for the first latent variable of the AUC and C_max_ prediction model, respectively; each dot represents a rat, plotted as the first latent variables (*X* block) vs. AUC or C_max_ (*Y* block). A color from blue to red represents the response variable from low to high. **(C)** and **(D)** are loading plots for the AUC and C_max_ prediction model, respectively. The blue box represents the response variable, each triangle represents a metabolite, and the triangles in red represent the metabolites with VIP >1.0.

Second, the intensities of 207 metabolites were correlated with AUC or C_max_ of Nuc in the initial PLS model to roughly investigate the relationship between the *X* and *Y* variables (Figure 6). The two-component PLS model is adopted for AUC and C_max_ prediction, which indicates a visible positive linear regression (Figure 6A, *R* ^2^ = 0.9824; Figure 6B, *R* ^2^ = 0.9797). Figures 6C, D indicate the loading plot of the aforementioned models and the relationship between predictive variable (*X*, triangle) and the response variable (*Y*, box). As shown in this loading plot, *X* variables on the top right or low left corner represent positive correlation to AUC or C_max_ and negative correlation to pharmacokinetic response variables. Besides, 88 (AUC) and 83 (C_max_) VIP >1.0 *X* variables were identified due to contribution of *X* variables to the PLS model (red triangles, Figures 6C, D), which were chosen for following prediction of AUC and C_max_, respectively.

### Prediction of AUC and C_max_ Based on Significant Metabolites

**FIGURE 7:**
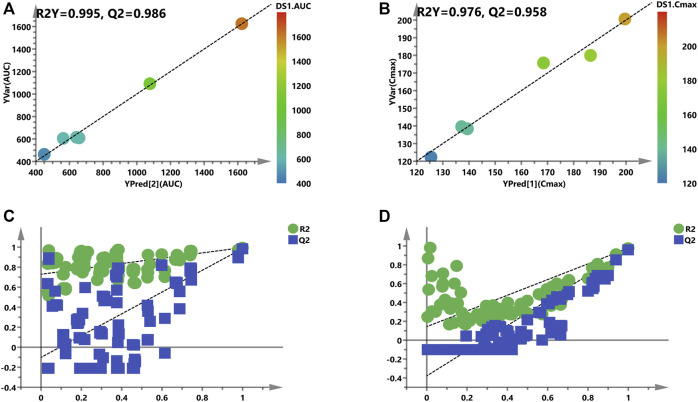
Refined models to predict individualized PK parameters based on the screened biomarkers. **(A)** and **(B)** are regression plots of the predicted PK parameters *vs.* the measured PK parameters (AUC or C_max_). The color from blue to red indicates the corresponding PK values from low to high. **(C)** and **(D)** are the resulting plots of the permutation test to identify the refined prediction models **(A)** and **(B)** without the risk of overfitting.

Considering the complex and difficult situation to predict PK parameters based on 88 and 83 variables, some significant and representative variables were screened for predictive biomarkers. Pearson’s correlation analysis was used for the association between PK parameters and VIP >1.0 variables. Regarding the prediction model, 16 or 12 VIP >1.0 screened variables were significantly correlated with AUC or C_max_, respectively (Table 1). Also, five common metabolites including adenosine, spermine, guanosine, sn-glycero-3-phosphocholine, and l-homoserine were found in both predictive models. As illustrated in Figure 7A, a PLS model was built based on the previous 16 variables, which helps explain about 99.5% variation (R^2^Y) and predict 98.6% variation (Q^2^) for the AUC. Simultaneously, as shown in Figure 7B, it could explain about 97.6% variation (R^2^Y) and predict 95.8% variation (Q^2^) in C_max_ based on the 12 variables. Permutation tests were performed with 100 iterations in order to avoid overfitting of this prediction model (Figures 7C, D). Overall, the results indicated that the prediction model exerted ability to predict AUC and C_max_ with no risk of overfitting. Variables listed in Table 1 were considered potential biomarkers for predicting AUC or C_max_.

**TABLE 1:** Potential predictive biomarkers for the pharmacokinetics of Nuc.

Model	Potential biomarker	VIP	Pearson coefficient*
AUC refined model	Adenosine	1.98	0.978^**^
	Spermine	1.94	0.956^**^
	Guanosine	1.92	0.946^**^
	3′,5′-cyclic AMP	1.86	0.919^**^
	Spermidine	1.83	0.903^*^
	DL 10:3	1.81	0.895^*^
	Reduced glutathione	1.80	0.887^*^
	sn-glycero-3-phosphocholine	1.78	−0.877^*^
	AMP	1.77	0.876^*^
	Guanosine 5′-monophosphate (GMP) disodium salt hydrate	1.77	0.875^*^
	IMP	1.77	0.874^*^
	l-homoserine	1.76	0.869^*^
	cAMP	1.76	0.869^*^
	Xanthine	1.74	0.857^*^
	Homocysteine	1.69	0.832^*^
	FA 16:0	1.67	−0.823^*^
C_max_ refined model	sn-glycero-3-Phosphocholine	2.05	−0.986^**^
	Guanosine	1.89	0.908^*^
	DL 18:0	1.88	0.903^*^
	Succinate	1.83	0.882^*^
	FA 12:0	1.82	0.878^*^
	FA 22:1	1.82	−0.876^*^
	l-homoserine	1.81	0.870^*^
	Adenosine	1.74	0.837^*^
	Niacinamide	1.74	0.838^*^
	Spermine	1.72	0.825^*^
	Thymidine	1.72	0.821^*^
	5α-cholanic acid-3,6-dione	1.69	−0.813^*^

*p < 0.05 and **p < 0.01.

**FIGURE 8:**
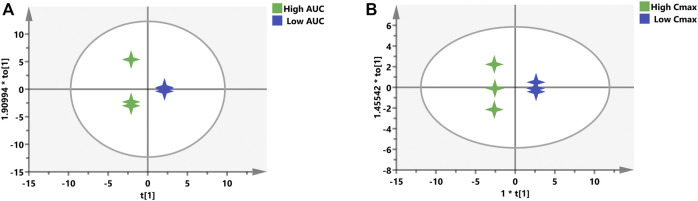
OPLS-DA models to discriminate the subgroups based on the screened biomarkers. Each 4-point star represents a rat. Green stars indicate rats with high AUC **(A)** or C_max_ **(B)**, while blue stars mean rats with low AUC **(A)** or C_max_ **(B)**.

For the purpose of validating this prediction ability of the aforementioned screened potential biomarkers, all rats were divided into high- and low-value groups according to their AUC and C_max_. Discrimination between high- and low-value groups based on the screened biomarkers was performed using OPLS-DA models. As described in Figure 8, the selected 16 biomarkers (AUC model) and 12 biomarkers (C_max_ model) completely distinguish the two groups, which indicate that these biomarkers are capable of discriminating drug exposure/response.

### Quality Control for Metabolomics

For the sake of obtaining reliable and reproducible results, several approaches and all sources of fluctuations have been identified and taken to minimize undesirable variation/bias, such as sample handling and preparation and HPLC-MS/MS system status. As shown in Supplementary Figure S3, the peak area ratios of pooled QC plasma samples were clustered under OPLS-DA which indicated that the fluctuation of pooled QC samples was small and constant pertaining to each analyte.

## Discussion

The main purposes of this research were to explore the targeted metabolic profiling after treatment by remdesivir in rats for better understanding the mechanism of remdesivir. To the best of our knowledge, no investigation is currently available regarding the metabolic perturbations and the relationship between metabolic fingerprint and remdesivir treatment. A total of 289 metabolites were included and analyzed for metabolic profiling using our previous method (Hu et al., 2020a; Hu et al., 2020b), which provided quantitation efficiency and high-throughput robustness completely (Figure 1). After careful chromatography peak double-checking by different experimenters, the raw data were imported to software for further statistical or descriptive analyses. Furthermore, both the longitudinal and transversal metabolomics of remdesivir was evaluated to reveal the metabolic trajectories, and reliable quality assurances through the present study guarantee high-quality data. It is noteworthy that metabolomics can not only provide a nearly instantaneous metabolite measurement but also maps specific metabolomes during normal or abnormal physiological condition, which renders metabolomics a powerful approach to assess response to drug, disease states, and especially short- and long-term metabolic effects mediated by infection and immunology (Nicholson et al., 2012; Diray-Arce et al., 2020; Du et al., 2020).

Regarding longitudinal metabolic analysis, metabolites in each rat almost assembled (Figure 2A). The most obviously disturbed metabolites, as shown in Supplementary Figure S4, included amino acid pathway (e.g., phenylacetylglycine, l-kynurenine, and l-cystathionine), carnitines (e.g., DL 5:1, DL 6:1, DL 11:0, and DL 10:3), cholic acids (e.g., deoxycholic acid/chenodeoxycholic acid, hyodeoxycholic acid, dioxolithocholic acid, and alloisolithocholic acid). Of note, these metabolites play a critical role in the physiological and pathological functions of SARS-CoV-2 (Asim et al., 2020). During the transversal metabolomics study, the metabolic profile was obviously distinguished between the pre-dose (baseline) and post-dose (drug treatment) group demonstrated by OPLS-DA. The permutation plot revealed the valid original model with all left Q^2^ values lower than right points, and the intersection of the regression line of the Q^2^-points and the vertical axis was less than zero (Figure 3). It has been reported that hyperinflammation with increased release of inflammatory cytokines is one of the critical characteristics, indicating cytokine storm in patients with COVID-19 (Mehta et al., 2020). Furthermore, McReynolds et al (McReynolds et al., 2021) reported an increased level of leukotoxin diols (metabolites from linoleic acid) in plasma samples of hospitalized patients suffering from severe pulmonary involvement. In this study, the linoleic acid metabolism pathway has the highest impact value, indicating that remdesivir treatment may disturb the metabolism of fatty acids to some extent. Amino acids (e.g., arginine, glutamine, glycine, proline, taurine, and tryptophan), peptides, and bioactive molecules have attracted more and more attention due to their abilities to reduce oxidative stress, inhibit apoptosis, and regulate immune responses (Chen et al., 2020). After treatment by remdesivir, most of these amino acids reduced compared with the baseline status. It has been reported that metabolites of kynurenate and kynurenine were enriched in COVID-19 patients, and more than 100 lipids (e.g., fatty acids and glycerophospholipids) were downregulated in COVID-19 patient sera (Shen et al., 2020). However, other publications indicated that the metabolism of tryptophan in the kynurenine pathway, which regulates inflammation and immunity, was increased in COVID-19 patients (Thomas et al., 2020).

In this metabolic fingerprint, most metabolites were downregulated owing to species difference (Figure 4) and disease status (healthy or baseline state and COVID-19 patients), and further studies are urgently necessary for comprehensively evaluating the metabolic profiles of remdesivir. Given the discrepancy of health or disease states, either larger external verification or virus-attacked status may be encouraging to further explore the metabolic disturbance. To date, no available investigation is reported regarding the interplay of metabolomics and pharmacokinetics. Thus, a correlation analysis was performed using multiple statistical approaches. It is reported that carnitines play a crucial role in the viral infection process. Bellamine et al. (Bellamine et al., 2021) revealed that l-carnitine tartrate supplementation in humans and rodents led to a significant decrease in angiotensin-converting enzyme 2 (ACE2), transmembrane protease serine 2, and Furin, which are in charge of viral attachment, viral spike S-protein cleavage, and priming for viral fusion and entry. Pretesting carnitine is necessary possibly to limit SARS-CoV-2 infection. In our study, dl-carnitine was significantly correlated with the concentration of Nuc (Figure 5), indicating the potential antiviral effect of Nuc *via* regulating metabolites of carnitine.

After checking for outliers, a two-stage PLS analysis including the initial and refined model was constructed between the metabolites and PK parameters (AUC and C_max_). The metabolic characteristics were significantly correlated with drug exposure (AUC and C_max_) to remdesivir, and several significant disturbance metabolites were found and further utilized for predicting the exposure of drug (Figure 6). Subsequently, these significantly changed metabolites were modeled and screened for potential predictive biomarkers. Finally, a total of 16 and 12 metabolites were selected and further validated (Figure 7). When COVID-19 ravaged the global health system, patients who developed interstitial pneumonia can evolve the inflammatory cytokine storm. As previously reported by academics, by means of its receptor, adenosine is capable to restrain the acute inflammatory process, enhance the protection capacity of the epithelial barrier, decrease the damage caused by the overactivation of the cytokine storms, and inhibit the adenosine transporters to decrease platelet activation and thrombosis (Falcone et al., 2020; Geiger et al., 2020; Caracciolo et al., 2021). Molecular docking analysis of the ACE-2 receptor protein also demonstrated that spermine phosphate has the maximum binding affinity and reactivity to ACE-2, indicating that spermine has great therapeutic potential in the treatment of COVID-19 (Mamidala et al., 2021). AT-511, the free base of AT-527 (an orally available double prodrug of a guanosine nucleotide analog), has potent stronger antiviral activity against SARS-CoV-2 *in vitro*, and the cytotoxicity was little at concentrations up to 100 μM in Huh-7 cells. These results suggested that AT-527 may be an effective therapeutic option against COVID-19 (Good et al., 2021). Moreover, based on these screened metabolites, an OPLS-DA model was used to further verify the predictive efficiency (Figure 8). Although limited samples are used in this study, several metabolite biomarkers were first discovered and provided to predict drug exposure/toxicity.

To date, no available investigation is reported regarding the comprehensive metabolic fingerprint after remdesivir treatment as well as correlation with PK parameters. Because of no definite therapeutic strategies for COVID-19, this study aims to explore the metabolomic characteristics of remdesivir and the potential biomarkers for predicting exposure or toxicity. Indeed, several limitations should be mentioned. First, the sample size of this exploratory research was small, and much data were needed to pool and verify these results. Accordingly, the external validation cohort should be replenished for better illustrating the comprehensive metabolomics. Second, due to the availability of blood samples from COVID-19 patients, we only investigated the metabolomics profile in rats. Taken together, our study first revealed the comprehensive metabolite trajectories induced by remdesivir and predictive drug exposure/toxicity biomarkers, which will provide a notable scientific contribution to prevention or therapy in patients with COVID-19.

## Conclusion

For the first time, we uncovered the comprehensive metabolic alterations after remdesivir treatment and revealed the potential predictive biomarkers for drug exposure or toxicity. Both longitudinal and transversal metabolic analyses were elucidated in rats after being administrated with remdesivir. Adenosine, spermine, guanosine, sn-glycero-3-phosphocholine, and l-homoserine may be considered potential biomarkers for predicting drug exposure or toxicity. Furthermore, this study is the first attempt to apply PM and PK to study drug response/toxicity, and the identified candidate biomarkers might be used to predict the AUC and C_max_, indicating the capability of discriminating good or poor responders to remdesivir treatment. Currently, this study originally offers considerable evidence to metabolite reprogramming and shed light on therapy development in fighting COVID-19.

## Data Availability

The datasets presented in this study can be found in online repositories. The names of the repository/repositories and accession number(s) can be found in the article/Supplementary Material.
